# Constant hydraulic supply and ABA dynamics facilitate the trade-offs in water and carbon

**DOI:** 10.3389/fpls.2023.1140938

**Published:** 2023-03-17

**Authors:** Mohanned Abdalla, Andreas H. Schweiger, Bernd J. Berauer, Scott A. M. McAdam, Mutez Ali Ahmed

**Affiliations:** ^1^ Department of Land, Air and Water Resources, University of California Davis, Davis, CA, United States; ^2^ Chair of Soil Physics, Bayreuth Center of Ecology and Environmental Research (BayCEER), University of Bayreuth, Bayreuth, Germany; ^3^ Department of Horticulture, Faculty of Agriculture, University of Khartoum, Khartoum North, Sudan; ^4^ Chair of Soil-Root Interactions, TUM School of Life Science, Technical University of Munich, Freising, Germany; ^5^ Institute of Landscape and Plant Ecology, Department of Plant Ecology, University of Hohenheim, Stuttgart, Germany; ^6^ Department of Botany and Plant Pathology, Purdue University, West Lafayette, IN, United States

**Keywords:** abscisic acid, drought, leaf age, leaf position, *Solanum lycopersicum* L., stomatal regulation, vapor pressure deficit

## Abstract

Carbon-water trade-offs in plants are adjusted through stomatal regulation. Stomatal opening enables carbon uptake and plant growth, whereas plants circumvent drought by closing stomata. The specific effects of leaf position and age on stomatal behavior remain largely unknown, especially under edaphic and atmospheric drought. Here, we compared stomatal conductance (*g_s_*) across the canopy of tomato during soil drying. We measured gas exchange, foliage ABA level and soil-plant hydraulics under increasing vapor pressure deficit (*VPD*). Our results indicate a strong effect of canopy position on stomatal behavior, especially under hydrated soil conditions and relatively low *VPD*. In wet soil (soil water potential > -50 kPa), upper canopy leaves had the highest *g_s_* (0.727 ± 0.154 mol m^-2^ s^-1^) and assimilation rate (*A*; 23.4 ± 3.9 µmol m^-2^ s^-1^) compared to the leaves at a medium height of the canopy (*g_s_*: 0.159 ± 0.060 mol m^2^ s^-1^; *A*: 15.9 ± 3.8 µmol m^-2^ s^-1^). Under increasing *VPD* (from 1.8 to 2.6 kPa), *g_s_*, *A* and transpiration were initially impacted by leaf position rather than leaf age. However, under high *VPD* (2.6 kPa), age effect outweighed position effect. The soil-leaf hydraulic conductance was similar in all leaves. Foliage ABA levels increased with rising *VPD* in mature leaves at medium height (217.56 ± 85 ng g^-1^ FW) compared to upper canopy leaves (85.36 ± 34 ng g^-1^ FW). Under soil drought (< -50 kPa), stomata closed in all leaves resulting in no differences in *g_s_* across the canopy. We conclude that constant hydraulic supply and ABA dynamics facilitate preferential stomatal behavior and carbon-water trade-offs across the canopy. These findings are fundamental in understanding variations within the canopy, which helps in engineering future crops, especially in the face of climate change.

## Introduction

Stomata regulate the exchange of water and carbon between plants and the atmosphere. Stomata adjust transpiration rate and plant hydration whilst controlling photosynthetic rate and plant growth. Stomatal regulation has been proposed to be a key feature allowing plants to rapidly respond to atmospheric and edaphic water deficits (Martin-StPaul et al., 2017), hereby impacting growth and productivity in natural and agricultural systems (Hetherington and Woodward, 2003). Despite these great importance, we are still far from fully understanding the mechanisms governing stomatal regulation under drought conditions (Buckley, 2019; Grossiord et al., 2020).

Various concepts have been proposed to understand and predict stomatal behavior. Carbon optimization theory, a pioneering concept predicting stomatal responses, posits stomata maximize carbon gain for a penalty of water loss (Cowan and Farquhar, 1977; Wang et al., 2020). Another approach suggests stomatal regulation restricts a decline in leaf water potential and soil-plant hydraulic conductance (Brodribb and McAdam, 2011; Buckley, 2019). Stomatal regulation in response to changes in leaf water status is believed to be actively controlled by the phytohormone abscisic acid (ABA), especially under stress conditions (e.g. Brodribb and McAdam, 2011; Merilo et al., 2018; Buckley, 2019). Combining the chemical and hydraulic signals was suggested to provide a holistic understanding of stomatal regulation (Buckley, 2019). Although there are recent attempts to reconcile different approaches to predict stomatal regulation during progressive soil drought (Joshi et al., 2022), the proposition of these various hypotheses indicates the challenges in understanding how stomata detect and react to intrinsic and extrinsic environments while maintaining plant water status (Brodribb and McAdam, 2011; Buckley, 2019).

Recent studies have endeavored to understand stomatal response to atmospheric and edaphic drought, namely, increasing vapor pressure deficit (*VPD*) and declining soil water content (Buckley, 2019; Lawson and Vialet-Chabrand, 2019; Brodribb et al., 2020; Grossiord et al., 2020). In response to increasing *VPD*, stomatal conductance increases briefly and decreases after prolonged exposure to high *VPD* (Buckley et al., 2011; Grossiord et al., 2020). Changes in leaf water potential and leaf hydraulic conductance have been assumed to facilitate guard cell responses to changes in *VPD* (Grossiord et al., 2020). On the other hand, stomatal response to soil water deficit can be explained within a hydraulic framework that highlights the capability of soil and plant to transport water under tension (Sperry and Love, 2015; Carminati and Javaux, 2020; Abdalla et al., 2022). For instance, the decline in soil-root hydraulic conductance has been recently documented as the main trigger of stomatal closure during soil drying (Rodriguez-Dominguez and Brodribb, 2020; Abdalla et al., 2021). Indeed, our understanding of the concomitant hydraulic capacities from soil to leaf under atmospheric and/or edaphic drought is, as yet, incomplete (Bartlett et al., 2016; Cuneo et al., 2016; Grossiord et al., 2020; Abdalla et al., 2022).

Despite these advances in understanding stomatal behavior, there are considerable uncertainties when scaling photosynthesis and stomatal conductance from leaf-scale to canopy-scale due to spatial variations within a plant canopy (Amthor, 1994; De Pury and Farquhar, 1997; Buckley, 2021). These variations are one of the main limitations stymieing the progress in understanding and predicting canopy scale stomatal responses to contrasting environments (Amthor, 1994; Buckley et al., 2014). Little is known about the response of leaves of different ages and positions to atmospheric and/or edaphic drought conditions.

Although variation in stomatal conductance within a plant crown has long been observed (Jarvis et al., 1976), the underlying mechanism governing this variation remain contentious. While there are numerous studies investigating the effects of leaf age on stomatal behavior in different species (Frank, 1981; Vos and Oyarzún, 1987; Bhagsari, 1988; Soar et al., 2004), there is limited research on the effect of leaf position. Buckley et al. (2014) suggested that carbon optimization theory alone cannot explain the variation in *g_s_* within the canopy (Buckley et al., 2014). More recently, Buckley (2021) used a mathematical model to illustrate that spatial variation in *g_s_* across the canopy could be explained by differences in irradiance. The interlinked effect of leaf age and position in the canopy on photosynthetic capacity, transpiration rate and leaf water potential remain under explored.

In this study, we addressed the knowledge gap regarding within canopy variation in *g_s_* regulation by investigating the effect of canopy position and leaf age on stomatal responses to edaphic and atmospheric droughts in tomato. We try to understand plant internal differences in age/position specific response differences not driven by the environment such as different degrees of light availability. We measured *g_s_*, *A* and transpiration rate (*E*) of different leaves of tomato across the canopy to increasing atmospheric drought (increasing *VPD*) under wet soil and during soil drying. We combined these measurements with those of foliage ABA level, canopy transpiration rate, soil moisture content, and soil water potential to better understand plant inherent-processes driving carbon-water tradeoffs apart from environmental effects across different leaf ages and positions across the canopy.

## Materials and methods

### Plant and soil preparations

We used tomato (*Solanum lycopersicum* L.) to perform our experiments, and we selected a variety (a hybrid with *Solanum pimpinellifolium*, Rootility^®^, Israel) that showed semi-determinate growth. The apical meristem was a vegetative one, however, side-branching occurred occasionally, which is suitable to test age and position specific effects. We also used the ABA-deficient mutant *flacca* and wild-type *Rhinelands Rhum*. The use of ABA deficient mutant and the corresponding wild type allow exploring the effects of ABA changes over time in response to increasing photosynthetic activity, especially in hydrated soils. Mutant plants were grown inside a humid chamber with the conditions of 90% RH and *ca.* 100 µm m^-2^ s^-1^ light intensity. These two genotypes were kept under high soil moisture and *g_s_* was measured in response to increasing light intensity from 0 to *ca*. 1000 µm m^-2^ s^-1^, stepwise. Seeds were surface-sterilized using 30% H_2_O_2_ for 60 seconds and germinated on Petri dishes containing wet filter paper for five days. Seeds were transplanted in PVC cylinders (30 cm in height, 9 cm in diameter). The sides of the cylinders had five holes, which were made every five centimeters to facilitate soil moisture measurements. The cylinders were filled with sandy loam soil, which was prepared by mixing quartz sand (37.5%) and loamy soil (62.5%). The substrates were sieved separately through a 1 mm sieve prior to mixing to achieve high degree of homogeneity among replicates. To measure soil water potential in each pot, a soil water potential sensor (Terros 21; Meter Group, Munich, Germany), with the dimensions of 9.5×3.5×1.5 cm, was buried in the middle (15 cm) of the cylinder.

### Growth conditions

Established seedlings were located inside a climate-controlled chamber with a day/night cycle at the temperature of 25/18°C, relative humidity of 62/67%, 12 hours of photoperiod with light intensity of 550 µmol m^-2^ s^-1^ (Luxmeter PCE-174, Meschede, Germany). Each plant was placed onto a balance, which routinely recorded the weight every 10 minutes. The soil surface was covered with polyolefin to prevent evaporation. Plants were daily irrigated for four weeks until the start of measurements. Plants were divided into two groups for measurements; three plants were subjected to a soil drying treatment where irrigation was withheld. Five plants were translocated to a laboratory (under similar ambient conditions and maintained in wet soil) to measure photosynthesis parameters.

### Soil dryness assessment

Before starting the experiments, the soil hydraulic properties (i.e., soil water retention and soil hydraulic conductivity) were measured, following the evaporative method, using Hyprop (Meter Group, Munich, Germany). This method evaluates changes in soil water content and soil water potential (at two depths) over time. Soil water retention curve was parameterized following Peters-Durner-Iden (PDI) model (Peters et al., 2015). The parameters were estimated by fitting the data points and solving Richards equation.

During soil drying experiment, soil water content was measured every day after the last irrigation using a time-domain refractometer (TDR; E-Test, Lublin, Poland). For each plant, soil water content was measured at five depths and the average value was considered as the soil water content. Soil water potential was additionally measured using water potential sensor (TEROS 21; Meter Group, Munich, Germany) during soil drying. The assessment of soil water content and soil water potential during soil drying treatments were presented in **Supplementary Figure S1**.

### Measurements of transpiration rate during soil drying

Inside the climate-controlled room, three plants were placed on wireless balances and automatically weighed every ten minutes. Canopy transpiration rate was obtained gravimetrically by calculating the differences of weight over the time course. We extracted midday transpiration rate (as the mean of transpiration rates between 12:00 and 13:00) for the days after last irrigation.

### Measurements of stomatal conductance during soil drying

Stomatal conductance, photosynthetic photon flux density, and leaf vapor pressure deficit were measured using a Li600 device (LI-COR Inc., Lincoln, NE, USA). For each plant, measurements were conducted at two canopy heights: a medium height 28 – 30 cm from the soil surface and in the upper canopy 57 – 61 cm above the soil, which was approximately equal to the total plant height. Three leaflets were measured at each canopy height per plant and each leaflet was measured three times. All leaf parameters were measured at the same time of the day (i.e., midday) during soil drying. Stomatal conductance, photosynthetic photon flux density, and vapor pressure deficit of the leaf were measured in three leaflets per canopy height, two heights per plant, and in a total of three plants. We calculated the mean value at each height (out of 3 leaflets that considered as technical replicates for high precision). Thus, error bars stand for variations among plants.

### Measurements of photosynthesis parameters

We measured photosynthesis parameters in different leaves, (1) young leaves (~10 days) at the top of the canopy, (2) young leaves (~8 days) at medium height and (3) fully expanded leaves (~15 days) at a medium height of the canopy. All selected leaves have been fully expanded and free from any damage. We measured the chlorophyll content using the chlorophyll-meter SPAD-502 Plus (Konica Minolta, Tokio, Japan) to ensure similarity in all groups of leaves and to exclude effects of leaf senescence. These measurements were conducted under wet soil conditions (soil water content = 0.23 ± 0.02 cm^3^ cm^-3^). Leaf gas exchange was measured using a LiCor6800 (LI-COR Inc., Lincoln, NE, USA). To quantify the response of leaf gas exchange to increasing atmospheric drought stress, we increased leaf *VPD* stepwise from 1 to 2.6 kPa (namely: 1, 1.4, 1.8, 2.2 and 2.6 kPa). The corresponding range of relative humidity was between 63.9% and 2.4%. Each step lasted 15 minutes and at a logging interval of 3 minutes. During the measurement, all other settings within the cuvette were hold constant at: CO_2_ reference 400 µmol mol^-1^, air temperature 22°C, photosynthetic photon flux density 1000 µmol m^-2^ s^-1^, fan 10.000 rpm and flow rate 500 µmol s^-1^. Leaf-level water use efficiency (WUE) was calculated by dividing *A* over maximum *E* of each leaf.

### Predicting assimilation rate

To simulate our measurements, we predicted *A* as a function of *g_s_* in the ABA-deficient mutant and the corresponding wild-type where it was not measured. According to Wankmüller and Carminati (2022), the relation between *A* and *g_s_* can be written following Michaelis-Menten equation


(1) $$A(gs)=\frac{\frac{gs}{1.6}A_{max}}{\frac{gs}{1.6}+K_{m}}$$

where *A*_max_ is the maximum measured assimilation rate, *K*_M_ is the Michaelis-Menten constant which is numerically equal to the *g_s_* at which *A* is half of *A*_max_ (Wankmüller and Carminati, 2022). The value of 1.6 is a conversion factor due to the different diffusivities of H_2_O and CO_2_ in the air (Tuzet et al., 2003).

### Measurements of leaf water potential

Leaf water potential was measured using a Scholander-type pressure chamber (Soil Moisture Equipment, CA, United States). After measuring leaf level gas exchange, the leaf was immediately cut and inserted in the leaf pressure chamber. Pressure was applied slowly and leaf water potential was determined when water appeared at the leaf-cut.

### Plant hydraulic conductance

Hydraulic conductance of the shoots (*K*_plant_) was determined at two levels of canopy’s height. Considering plant as a porous medium, we applied Darcy’s law to obtain *K*_plant_ as follow:


(2) Kplant=EΔψ

where, *E* is the transpiration rate (mmol m^-2^ s^-1^), and Δψ was the pressure gradients across the soil-plant system (MPa). In this study, the gradient from main stem to the leaf surface is the crucial component of *K*_plant_ due to the similar below-ground conditions.

### ABA content measurements

Leaf samples were collected after measuring leaf water potential and stored in methanol at -20°C. The part of the leaf that was enclosed inside the IRGA chamber (Licor Li6800) was collected separately and used for ABA quantification. The plant material was grounded in liquid nitrogen and then weighed (ca. 30 mg fresh weight) into 2 mL plastic micro-tubes (Eppendorf AG, Germany). Before extraction, two 3 mm ceria-stabilized zirconium oxide beads were placed into each tube. The samples were extracted and purified after Šimura et al. (2018). For phytohormone extraction, 1 ml ice-cold 50% aqueous (v v^-1^) acetonitrile (CAN) containing the internal standards was added to each tube. Deuterated ABA ([2H6] (+)-cis,trans-Abscisic Acid, olchemim ref: 034 272x) and Deuterated PA ((-)-7’-7’-7’-d3-Phaseic Acid, NCR) were used as internal standards with the concentration of 5 nM per 1 ml. All samples were purified using Oasis PRIME HLB RP (1 cc per 30 mg), polymer-based SPE cartridges (Waters Co., USA). Afterward, the samples were evaporated to dryness at 40°C in a vacuum concentrator RVC 2-33 IR (Martin Christ GmbH, Germany) and stored at −20°C until analysis. For analysis, the samples were dissolved in 50 µl of 30% ACN (v v^-1^) containing 0.1% FA and transferred to insert-equipped vials. The absolute quantification of targeted phytohormones was performed by UHPLC-HESI-HRMS. Separation of detected compounds was achieved on a reversed phase Acquity UPLC^®^ HSS T3 column (10 Å, 2.1 × 100 mm, 1.8 μm, Waters) using a gradient elution of A (Water, 0.1% FA) and B (ACN, 0.1% FA) as follows: 0–5 min, 10% B; 5–10 min, 10% to 80% B. The injection volume was 5 μl. The UHPLC system was coupled to a Q Exactive Plus Mass Spectrometer (San Jose, CA, USA) equipped with a HESI source operating in negative ion mode. To generate the calibration curve, the peak area on the extracted ion chromatogram (XIC) of the deprotonated molecule ion [M-H]- was measured. A least-square linear regression was used to best fit the linearity curve.

### Statistical analyses

For the VPD-response experiment, we tested the relationships between assimilation rate (*A*), transpiration (*E*), stomatal conductance (*g_s_*) and vapor pressure deficit (*VPD*) using linear regression models. We used simple linear relationships as well as quadratic and logarithmic transformations of the predictors (*VPD* and *g_s_*). We used comparisons of model diagnostics (QQ-plots) and measures of fit (adjusted R^2^) to choose the most appropriate predictor transformation. Effects of leaf position and leaf age were tested by including leaf position and leaf age as additional explanatories in the regression models. Effects of leaf age were tested by comparing responses of young and old leaf measured in the same, middle position of the plants in a multiple linear regression model with leaf age as an additional, factorial explanatory. Leaf position effects were tested by comparing young, fully expanded leaves from the top of the plants with young, fully expanded leaves from the middle position of the investigated plants and by putting leaf position as an additional, factorial explanatory factor in the regression model. Relative importance of leaf position and age in explaining differences in the observed patterns was quantified by variance partitioning of each set of leaves using the *vegan* R package (v.2.5-6, Oksanen et al., 2019).

We analyzed the relative effect of age (mid-young *vs.* mid-old) and position (mid-young *vs.* top-young) in the response of leaf gas exchange parameters (i.e. *A*, *E*, *g_s_*) to increasing *VPD*. To do so, we calculated the absolute difference in relative increment per increase in *VPD*. In detail, we first calculated the mean of each gas exchange parameter at the equilibrium (last measurement) of each *VPD* step for each of the three leaf age and leaf position combinations. Second, the relative change between x[t] and x[t-1] was quantified, followed by subtracting the difference between comparison pairs for age- respectively position effect from 1 Thus, the closer to 1 the more similar the response to increasing *VPD* is.

For the soil drying experiment, we used two-way analysis of variance (ANOVA), followed by multiple comparison, to test the significance of leaf position and soil drying and their interactions on stomatal conductance. One-way ANOVA was used to test the differences in ABA content, leaf water potential and plant hydraulic conductance among different groups of leaves, independently. All analyses were performed in R (v. 3.6.1, R Core Team, 2019) and MATLAB (Math Works Inc., USA) using a level of significance of α=0.05.

## Results

**Figure 1:**
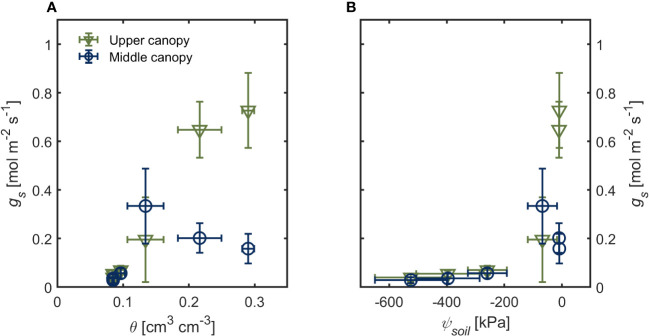
Stomatal conductance (*g_s_*) of upper canopy leaves (green triangles) and leaves in the middle canopy (blue open symbols) during soil drying. **(A)** soil water content (*θ*) and soil **(B)** water potential (*ψ*_soil_). Error bars stand for the standard deviation, and *n* = 3.

Under hydrated soil conditions (*θ* > 0.20 cm^3^ cm^-3^ *ψ_soil_* ~ -10 kPa), upper canopy leaves had four times higher *g_s_* (0.727 ± 0.154 mol m^-2^ s^-1^) than lower-canopy leaves (0.159 ± 0.060 mol m^-2^ s^-1^; **Figure 1A**; *p*< 0.001; **Supplementary table S1**). This difference in *g_s_* across the canopy was only apparent under wet soil conditions (**Figure 1**). As soil moisture started to decrease (*θ* ≤ 0.13; *ψ_soil_* < -50 kPa), we observed no differences in *g_s_* across the canopy (**Figures 1A, B**). Stomatal conductance (*g_s_*) declined steeply as soil water potential declined (**Figure 1B**; *p* < 0.001; **Supplementary table S1**). There were no differences in PPFD between the two canopy positions during the drying cycle (402.91 ± 29.89 μmol m^2^ s^-1^ and 401.98 ± 13.51 μmol m^2^ s^-1^ for upper and lower canopy leaves, respectively; **Supplementary Figure S2**). Leaf vapor pressure deficit (*VPD_leaf_*) was 0.8 kPa in the first three days after last irrigation (when the soil was wet; *θ* > 0.13) and increased afterward to more than 1.2 kPa in dry soils (*θ* < 0.10; **Supplementary Figure S3**). Canopy transpiration rate followed similar trends as *g_s_* during soil drying (*θ* < 0.13; *ψ_soil_* < -50 kPa; **Supplementary Figure S4**).

**Figure 2:**
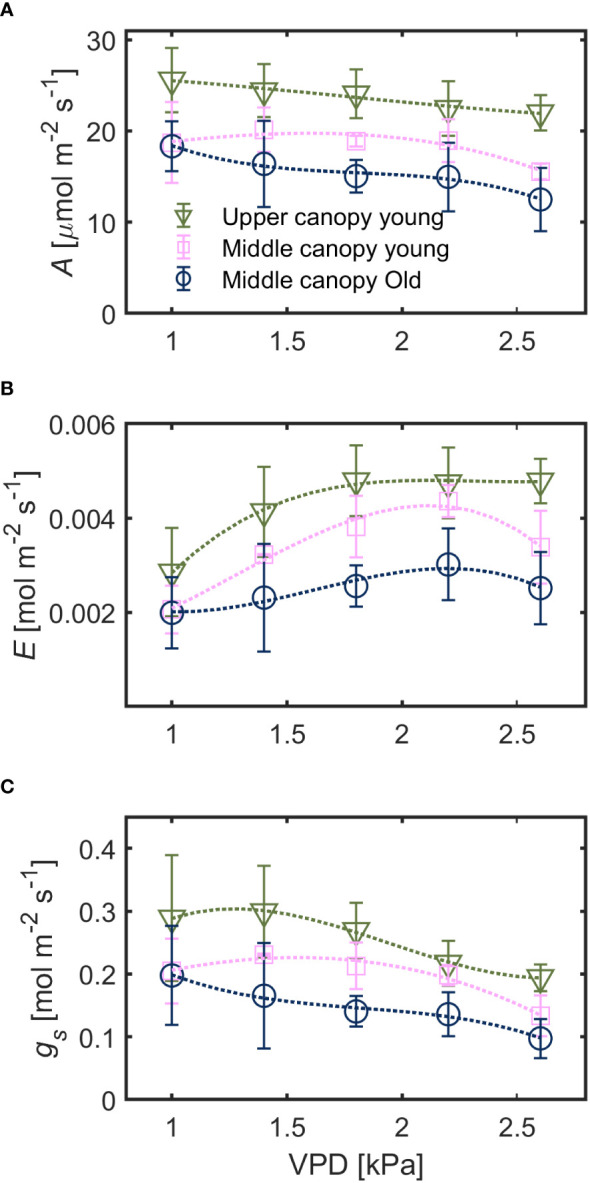
Responses of **(A)** assimilation rate (*A*), **(B)** transpiration rate (*E*) and **(C)** stomatal conductance (*g_s_*) to increasing vapor pressure deficit (*VPD*) in leaves with spatial and ontogenetic variations, under hydrated soil conditions. Green triangles for upper canopy leaves, pink squares for middle canopy young leaves and blue open symbols for middle canopy old leaves across panels. Dashed lines are predictions of a regression model. Error bars stand for the standard deviation, and *n* = 5.

Under hydrated soil conditions, leaves at different canopy positions exhibited a contrasting stomatal sensitivity to changes in *VPD* (**Figure 2**). Net assimilation rate (*A*) was highest for upper canopy young leaves (23.4 ± 3.9 µmol m^-2^ s^-1^) followed by middle canopy young leaves (18.4 ± 3.5 µmol m^-2^ s^-1^) and middle canopy old leaves (15.9 ± 3.8 µmol m^-2^ s^-1^; effect of leaf identity *p*< 0.001 based on robust linear mixed effect model with *VPD* as random effect). Leaf-level water use efficiency (*WUE*) was lowest for upper canopy young leaves (0.59 ± 0.24 µmol mol^-1^) and highest for middle canopy old leaves (0.67 ± 0.28) with middle canopy young leaves showing intermediate *WUE* (0.64 ± 0.33, effect of leaf identity *p*< 0.001 based on a linear mixed effect model with *VPD* as random effect). Both *A* and *g_s_* generally decreased with increasing *VPD* while transpiration rate (*E*) increased across all leaves (**Figure 2**). Variation in *A* of young leaves (mid *vs.* top) was mainly explained by canopy position (26% of total variation) and to a minor degree by variation in *VPD* (7%). Assimilation of middle canopy leaves (young *vs.* old, non-senescent) was explained by leaf age and *VPD* to a similar degree (8%, each). For the relationship between stomatal control/water loss (*E*, *g_s_*) and atmospheric water demand (*VPD*), the effect of canopy position was comparable to the effect of leaf age for transpiration rate (9%). Canopy position was more important than leaf age for explaining differences in *g_s_* (11% *vs.* 5%).

**Figure 3:**
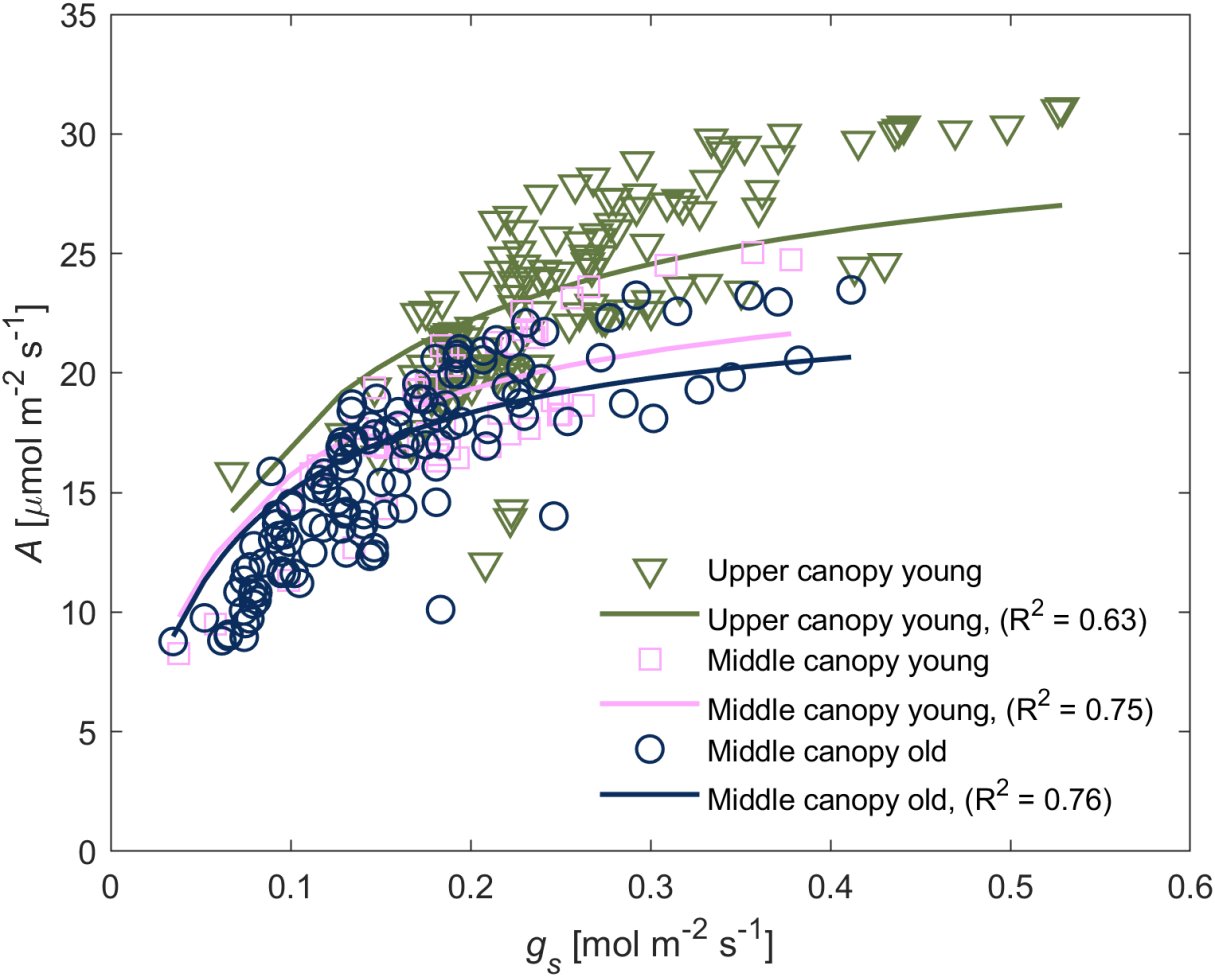
Relationship between carbon assimilation rate (*A*) and stomatal conductance (*g_s_*) of leaves from different canopy height and age. The *A*(*g_s_*)-relation varied between different groups. The relationship was reproduced through Michaelis-Menten saturation curve. Symbols represent measurements whereas solid lines represent the fitted model using Eq. (1), and the goodness of the fitting is indicated within the respective panel.

**Figure 4:**
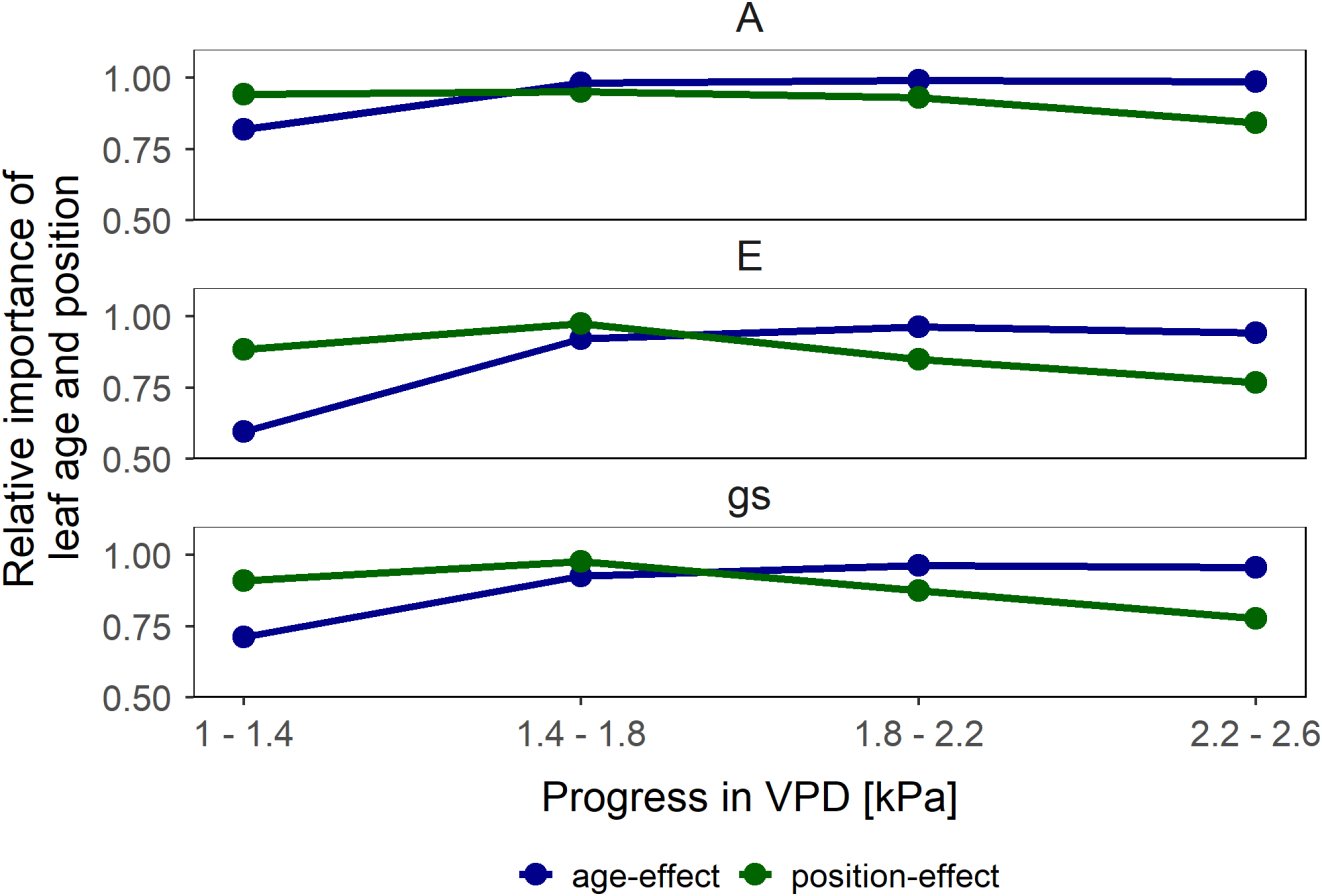
Relative importance of leaf age (similar canopy position but different age in blue) and canopy position (different position but similar age in green) effects on leaf gas exchange (assimilation rate (*A*), transpiration rate (*E*) and stomatal conductance (*g_s_*)). Vapor pressure deficit (*VPD*) alters the relative importance of age and canopy position on leaf gas exchange parameters. Symbols represent the mean difference in the relative change between the respective comparison pairs subtracted from 1.

We observed a positive relationship between *A* and *g_s_* in wet soil (**Figure 3**). For young leaves, *g_s_* explained the highest portion of variation of *A* (47% of total variation) whereas canopy position as an individual explanatory variable explained only a minor degree of the variation (5%). Similarly, *A* in middle canopy leaves was predominantly explained by *g_s_* (70%) whereas leaf age as an individual factor was irrelevant (0%). The simplified model predicted the differences in *A* among different groups of leaves. *A*, *E* and *g_s_* at low *VPD* – between 1.0 and 1.8 kPa – was predominantly influenced by canopy position, whereas at high *VPD* – between 1.8 and 2.4 kPa – the leaf age became the dominant factor (**Figure 4**).

**Figure 5:**
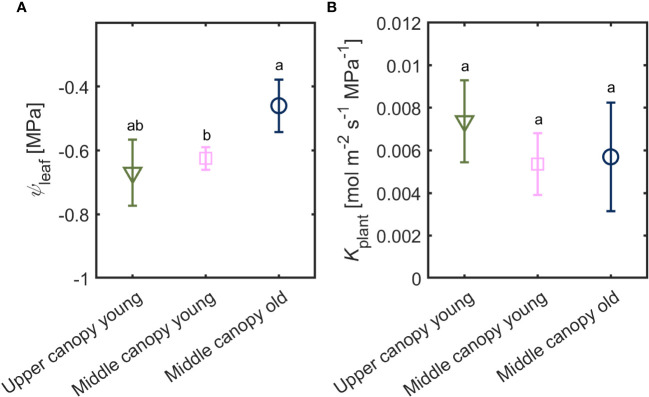
**(A)** Leaf water potential (*ψ*_leaf_) and **(B)** plant hydraulic conductance (*K*_plant_) of leaves from different canopy height and age (*n* = 5).

**Figure 6:**
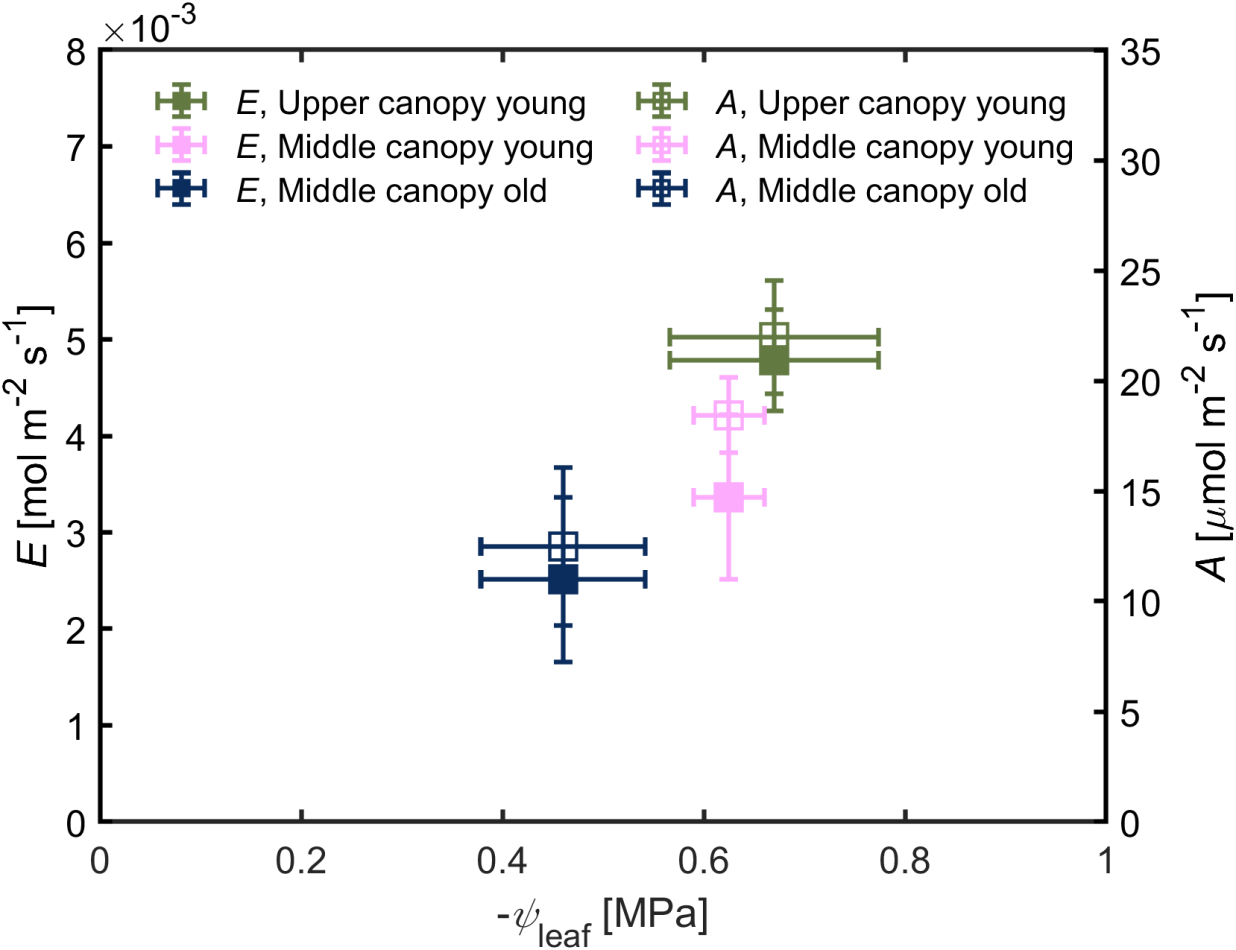
Transpiration rate (*E*) and carbon assimilation rate (*A*) as a function of leaf water potential (*ψ*_leaf_). Green triangles for upper canopy leaves, pink squares for middle canopy young leaves and blue open symbols for middle canopy old leaves. Error bars stand for the standard deviation, and *n* = 5.

Leaf water potential was slightly lower (-0.67 ± 0.10 MPa) in leaves located on the upper canopy in comparison to middle canopy leaves (-0.46 ± 0.08 MPa; **Figure 5A**). Middle canopy young leaves had a lower leaf water potential (-0.625 ± 0.04 MPa) compared to middle canopy old leaves (**Figure 5A**; *p*< 0.05; *n* = 5; **supplementary table S2**). Plant hydraulic conductance was constant across age and canopy position (**Figure 5B**; *p* = 0.41; *n* = 5; **Supplementary Table S3**). At VPD of 2.6, high *A* and *E* were associated to more negative leaf water potential in upper leaves, while lower *A* and *E* were correlated with less negative leaf water potential in middle canopy leaves (**Figure 6**).

**Figure 7:**
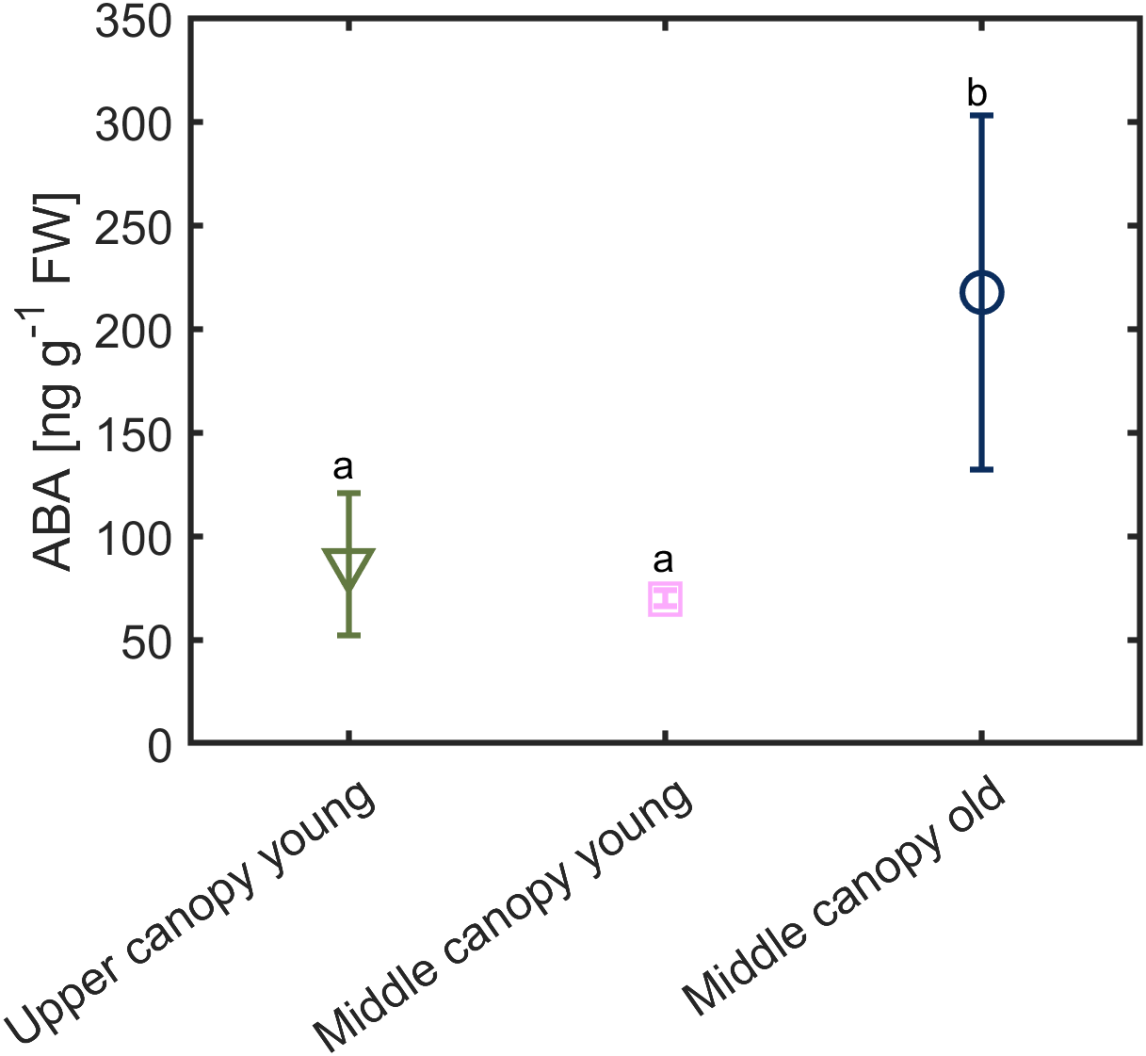
Foliage ABA level in across the canopy exposed to relatively high vapor pressure deficit (VPD = 2.6 kPa) under hydrated soil conditions (soil water content = 0.23 ± 0.02 cm^3^ cm^-3^). Different letters denote significant difference in ABA contents. Error bars stand for the standard deviation (*n* = 5).

Under hydrated soil conditions and high VPD, we observed a difference in the foliage ABA level across the canopy (**Figure 7**). The ABA level was twice as high in middle canopy old leaves (217.56 ± 85 ng g^-1^ FW) compared to upper canopy leaves (85.36 ± 34 ng g^-1^ FW) and middle canopy young leaves (69.94 ± 3.96 ng g^-1^ FW; *p* = 0.0281, *n* = 6; **Supplementary Table S4**).

**Figure 8:**
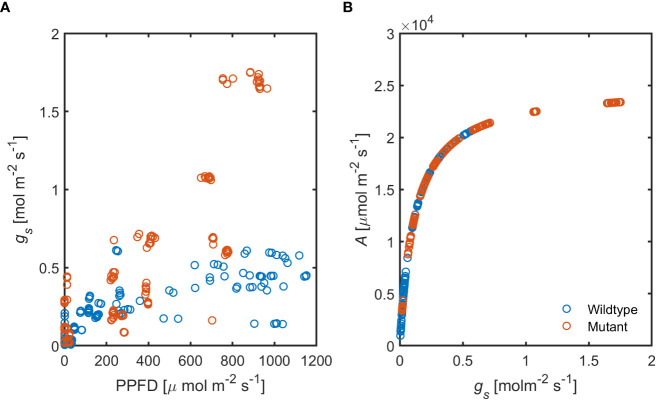
Relationship between increasing photosynthetic activity and stomatal conductance, **(A)** in the ABA-deficient mutant (red) wild-type (blue) plants. **(B)** Predicted assimilation rate (*A*) in response to measured *g_s_* in both genotypes (*n* = 6).

Stomatal conductance (*g_s_*), in response to increasing photosynthetic activity, was substantially higher in the ABA-deficient mutant compared to the wild-type (**Figure 8A**). These data show that ABA level plays a substantial role in stomatal regulation under hydrated soil conditions and high PPFD.

## Discussion

Functional differences were observed in stomatal behavior across the canopy. Although the effect of leaf age on stomatal conductance is well documented and discussed to be due to intrinsic variations in photosynthetic capacity (Vos and Oyarzún, 1987; Atkinson et al., 1989; Soar et al., 2004; Chen et al., 2013), little is known about the relative importance of leaf age *vs.* leave position in driving carbon-water trade-offs. Our results indicate a strong impact of canopy position on stomatal behavior, especially under hydrated soil conditions and relatively low VPD. Yet, the modulating effect of canopy position *vs*. leaf age changed with increasing atmospheric drought, which induced higher levels of ABA in old leaves compared to young leaves, regardless of their positions, even under wet soil conditions (**Figure 7**; McAdam et al., 2022). Furthermore, the high stomatal conductance of upper canopy leaves vanished as soil water potential declined (**Figure 1**).

A stable root, soil and leaf hydraulic conductance, under hydrated soil conditions, facilitated proportionality between the gradients in leaf water potential and the increment in transpiration rates across the canopy (**Figures 2**, **3**, **5** and **6**). Similar trends of constant hydraulic supply have also been documented, under ample soil water contents, in tomato (Abdalla et al., 2021; Abdalla et al., 2022), as well as other crops and trees (Cai et al., 2020; Bourbia et al., 2022; Cai et al., 2022). Moreover, in tomato, xylem tissues of root, stem and leaves have similar vulnerability to embolism under water stress conditions (Skelton et al., 2017), which entails similar water transport capabilities under water deficit. Constant hydraulic supply makes it possible for upper canopy leaves to sustain higher evaporation rates (**Figure 6**).

Although all measured leaves had similar chlorophyll content, we observed difference across ages and canopy position in gas exchange parameters (**Figure 3**). One possible explanation for these differences is the contrasting levels of phytohormones across the canopy (e.g., ABA; **Figure 7**) together with the slight gradients in leaf water potential (**Figure 5A**). The correlation between hydraulic and chemical signal has been proposed to control stomatal regulation (Buckley, 2019). Sack et al. (2018) concluded that any factor causes a decline in leaf water content (subsequently leaf water potential) will increase ABA synthesis, especially under rapid changes in vapor pressure deficit, which was the case in this study. This conclusion is in line with our finding that ABA played a prominent role in regulating stomata under high VPD and wet soil conditions (**Figure 8**). Measuring *g_s_* in response to increasing photosynthetic photon flux density in ABA-deficient mutant and the corresponding wild type made it possible to explore the impacts of ABA dynamics on the relationship between stomatal conductance and assimilation rate. Our simple simulation of *A*(*g_s_*) relationship, in the ABA-deficient mutant and the corresponding wild-type, suggests a correlation between photosynthetic activity and ABA dynamics (**Figure 8**). Additionally, recent evidence has shown that ABA is mainly produced in leaves (McAdam and Brodribb, 2018), and root sourced ABA has been shown to play no role in stomatal regulation in tomato (Holbrook et al., 2002). Furthermore, Wankmüller and Carminati (2022) proposed a correlation between ABA content, the decline in leaf water potential and the photosynthetic activity.

Under high VPD and hydrated soil conditions, we observed high ABA levels and less negative leaf water potential in old *vs.* young leaves in the middle canopy. A possible explanation is that high ABA content in old leaves induces stomatal closure, reducing transpiration rate and water loss, hence preventing the leaf from reaching critically low (more negative) leaf water potential (Wankmüller and Carminati, 2022). Low ABA content in young leaves is associated with higher stomatal conductance and more negative leaf water potential (McAdam et al., 2022). This phenomenon allows young leaves to exhibit higher stomatal conductance and to explore more negative leaf water potential to acquire more carbon under wet soil conditions (**Figures 6**–**8**). Thus, this explains the lower impacts of ABA on leaves in the apical parts of the canopy compared to fully expanded leaves located on a medium height, which is in line with the findings of Soar et al. (2004), who observed spatial gradients of ABA along canes of grapes (*Vitis vinifera* L.).

Non-stomatal limitations might have impacted the relationship between assimilation rate and stomatal conductance in different groups of leaves. Variations in CO_2_ diffusion, within the mesophyll, among different leaves, can directly impact leaf photosynthetic capacity at a given stomatal conductance. Different photosynthetic capacities can be due to variations in biochemical reactions, such as the ratio of chlorophyll-a to chlorophyll-b, the efficiency of rubisco carboxylation or electron transport rate across the plasmatic membrane (von Caemmerer and Farquhar, 1981). Additionally, the atmospheric conditions directly at the leaf surface might have additional effects on nitrogen content and leaf morphology, leaf mass per unit area and cuticle thickness (Weerasinghe et al., 2014). These factors and other non-stomatal limitations to photosynthesis and should be considered to fully understand leaf gas exchange under constant hydraulic supply.

Differences in stomatal regulation across the canopy occurred only under ample water conditions, and stomatal conductance decreased rapidly as soil water potential declined regardless of canopy position and age (**Figure 1**). In dry soil conditions, root, soil and/or rhizosphere hydraulic conductance has been reported as the main hydraulic limitation across the soil-plant continuum triggering stomatal closure (Rodriguez-Dominguez and Brodribb, 2020; Abdalla et al., 2021; Bourbia et al., 2021). This hydraulic bottleneck might hinder preferential stomatal conductance within the canopy, suppressing the canopy position effect in dry soils by restricting the water fluxes from the soil (**Figure 1**). The premise is that water potential dissipation in soil causes a drop in the hydraulic conductivity around roots. In contrast to the plant hydraulic conductivity, soil hydraulic conductivity decreases by several orders of magnitude during drying (Passioura, 1988; Draye et al., 2010; Cai et al., 2022). Taken together, during the vegetative stage, soil drying obliterates the effect of leaf position on stomatal conductance and consequently assimilation rate, owing to the fact that preferential stomatal conductance occurred only under wet conditions.

This study provides direct evidence that ABA plays a pivotal role in stomatal regulation in response to high VPD (McAdam and Brodribb, 2016; Buckley, 2019; Lawson and Vialet-Chabrand, 2019), even under hydrated soil conditions (**Figure 8**). We observed a stable hydraulic conductance from soil to leaf under increasing transpiration demand in hydrated soil conditions. The stable water supply allowed the actively growing leaves, under ambient soil water conditions, to maximize carbon uptake hence increasing the assimilation rate in apical and/or juvenile leaves, while losing water. Safety-efficiency trade-offs in water loss and the decline in leaf water potential were documented in several plant species (Henry et al., 2019). Here, we reported trade-offs in carbon and water balance on plant-scale, which were facilitated by the constant hydraulic conductance across the soil-leaf system and ABA levels. Thus, in hydrated soil conditions, plants were capable of mitigating the effect of atmospheric drought, especially within the measured range. Further investigations are needed on the interactions of edaphic drought and light competition in different plant species. We additionally conclude that soil drought, by suppressing preferential stomatal behavior, could impede the competitive strength of plant species with differed preferential stomatal conductance, hence changing the competitive structure of entire plant communities (Hautier et al., 2009). On the other hand, mechanisms that maintain constant hydraulic supply across soil-plant system, especially during soil drying, could provide a great advantage in growth and development of a species (Abdalla et al., 2021; Bourbia et al., 2022; Harrison Day et al., 2022). Such mechanisms could be a long and dense root system with low hydraulic conductance (Abdalla et al., 2022; Cai et al., 2022), long and dense root hairs (Carminati et al., 2017; Marin et al., 2021), plasma membrane aquaporin (Caldeira et al., 2014), root mucilage (Ahmed et al., 2014; Carminati et al., 2016) and/or root symbiosis with arbuscular mycorrhizal fungi (Bitterlich et al., 2018; Abdalla and Ahmed, 2021). The trade-offs between water and carbon might have furthermore important implications on the growth vigor of juvenile plants and the establishment of crops growing in contrasting environments.

## Data availability statement

The original contributions presented in the study are included in the article/**Supplementary Material**. Further inquiries can be directed to the corresponding author.

## Author contributions

Conceptualization: MoA, AS, BB, SM and MAA. Data curation during soil drying: MoA. Data curation during atmospheric drying: AS, BB, MoA and MAA. Data analysis: MoA, BB and AS. MoA wrote the manuscript with contributions from all authors. All authors contributed to the article and approved the submitted version.
