# Supplementary material for: Constant hydraulic supply and ABA dynamics facilitate the trade-offs in water and carbon
Source: Front Plant Sci. 2023 Mar 17;14:1140938. doi: 10.3389/fpls.2023.1140938 (PMC10064056; doi:10.3389/fpls.2023.1140938)
Supplement: Supplementary file 1 [file DataSheet_1.pdf]

Constant hydraulic supply and ABA dynamics facilitate the trade-offs in water and carbon

Mohanned Abdalla, Andreas H. Schweiger, Bernd J. Berauer, Scott A. M. McAdam, and Mutez Ali Ahmed

Supplementary information

Supplemental Table S1 Two-way analysis of variance (ANOVA) considering the influences of leaf position and soil drying and their interactions on stomatal conductance.

Source	Sum Sq.¶	d. f.	Mean Sq.	F	Prob. > F
**Leaf position**	0.816	1	0.816	99.23	**<< 0.0001*** **
**SWC¶¶**	3.291	4	0.822	99.94	**<< 0.0001*** **
**Leaf position × SWC**	1.806	4	0.451	54.87	**<< 0.0001*** **
**Error**	0.806	98	0.008		
**Total**	6.537	107			

¶ **Sum Sq**.: sum of squares, **d. f**.: degree of freedom, **Mean Sq.:** Mean sum of squares, **F**: F-statistic value. $p < 0.001$***, $p < 0.01$**, $p < 0.05$*.

¶¶ SWC: soil water content, was considered in five levels (0.29; 0.21; 0.13; 0.09; 0.08).

Supplemental Table S2 One-way analysis of variance (ANOVA) identifying any significant differences in leaf water potential between groups of leaves.

Source	Sum Sq.¶	d. f.	Mean Sq.	F	Prob. > F
**Leaves groups**	0.116	2	0.0581	7.43	**0.0129***
**Error**	0.071	9	0.0079		
**Total**	0.187	11			

¶ **Sum Sq**.: sum of squares, **d. f**.: degree of freedom, **Mean Sq.:** Mean sum of squares, **F**: F-statistic value. $p < 0.001$***, $p < 0.01$**, $p < 0.05$*.

Supplemental Table S3 One-way analysis of variance (ANOVA) identifying any significant differences in plant hydraulic conductance between the groups of leaves.

Source	Sum Sq.¶	d. f.	Mean Sq.	F	Prob. > F
**Leaves groups**	9.27×10^{-6}	2	4.63×10^{-6}	0.97	0.41
**Error**	4.29×10^{-5}	9	4.77×10^{-6}		
**Total**	5.22×10^{-5}	11			

¶ **Sum Sq**.: sum of squares, **d. f**.: degree of freedom, **Mean Sq.:** Mean sum of squares, **F**: F-statistic value. $p < 0.001$***, $p < 0.01$**, $p < 0.05$*.

Supplemental Table S4 One-way analysis of variance (ANOVA) identifying any significant differences in ABA content between the groups of leaves.

Source	Sum Sq.¶	d. f.	Mean Sq.	F	Prob. > F
**Leaves groups**	45172.2	2	22586.1	6.22	**0.0281***
**Error**	25422.3	7	3631.8		
**Total**	70594.5	9			

¶ **Sum Sq**.: sum of squares, **d. f**.: degree of freedom, **Mean Sq.:** Mean sum of squares, **F**: F-statistic value. $p < 0.001$***, $p < 0.01$**, $p < 0.05$*.

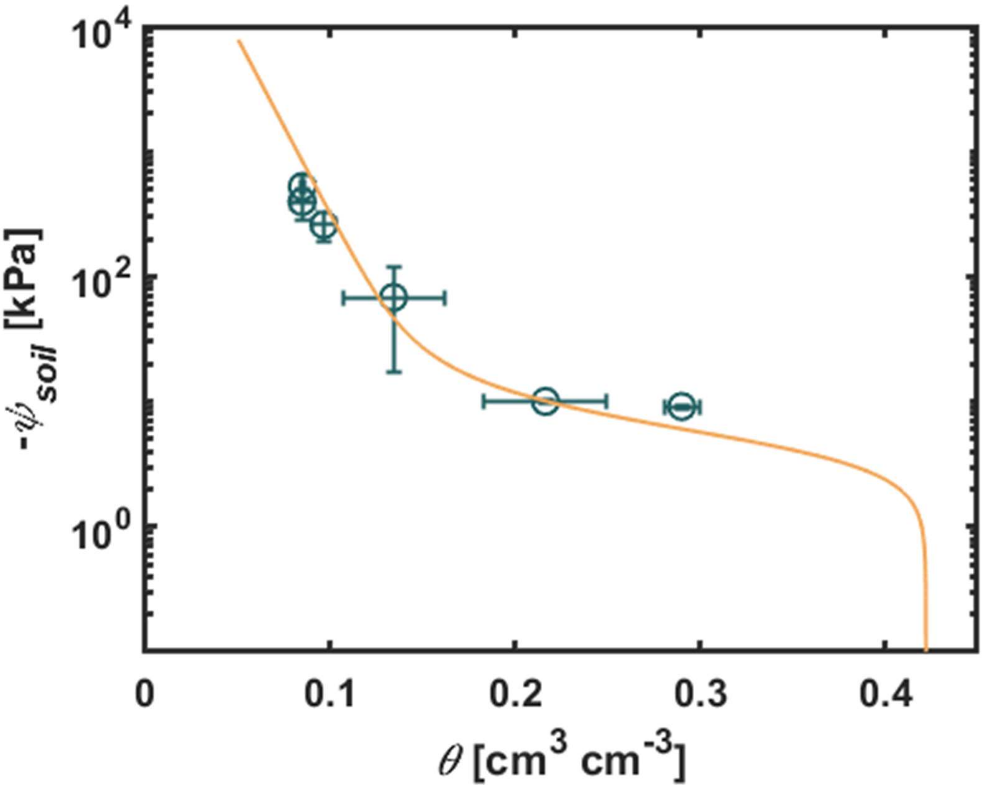

Fig. S1. Soil water retention curve as measured with evaporation method and parameterized with PDI model (orange line). Green open symbols mark daily measurements of soil water potential (ψ_{soil}) and soil water content (θ) during the experiment. Soil water content was measured with the TDR, and soil matric potential was measured with the matric potential sensor (Terros 21).

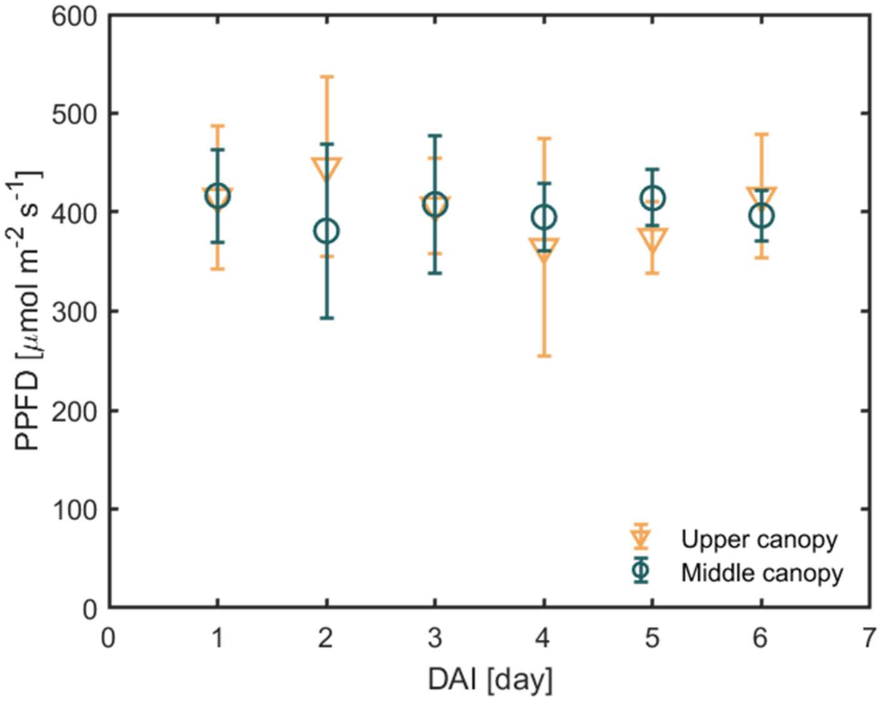

Fig. S2. Photosynthetic photon flux density (PPFD) as measured on upper canopy leaves (orange triangles) and middle leaves (green open symbols). No differences in PPFD between the two leaves' positions during the drying cycle (DAI: day after last irrigation).

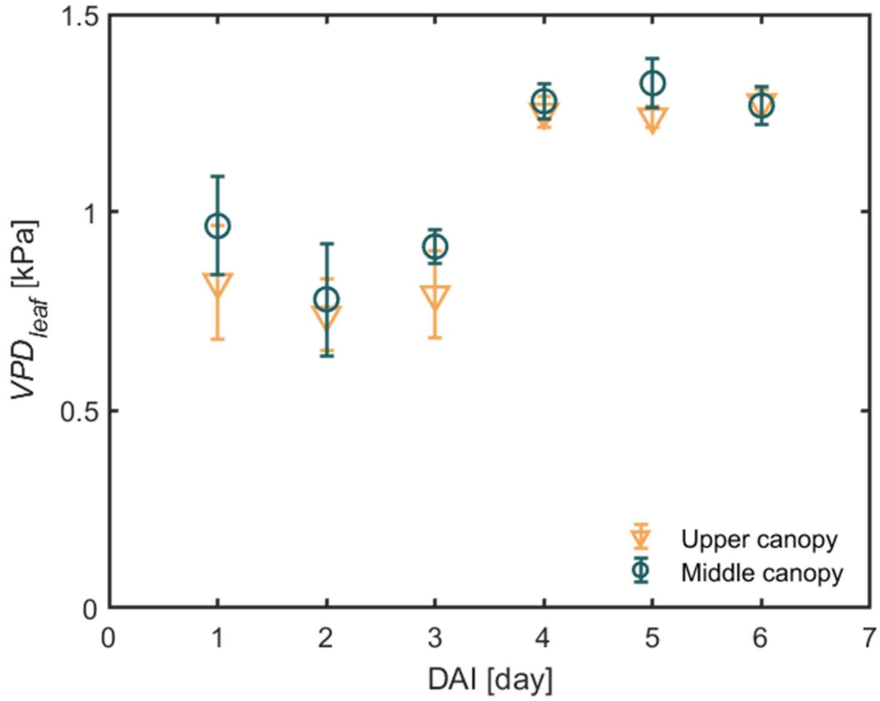

Fig. S3. Leaf vapor pressure deficit (*VPD_{leaf}*) was low in the first three days after last irrigation (DAI) and increased afterward.

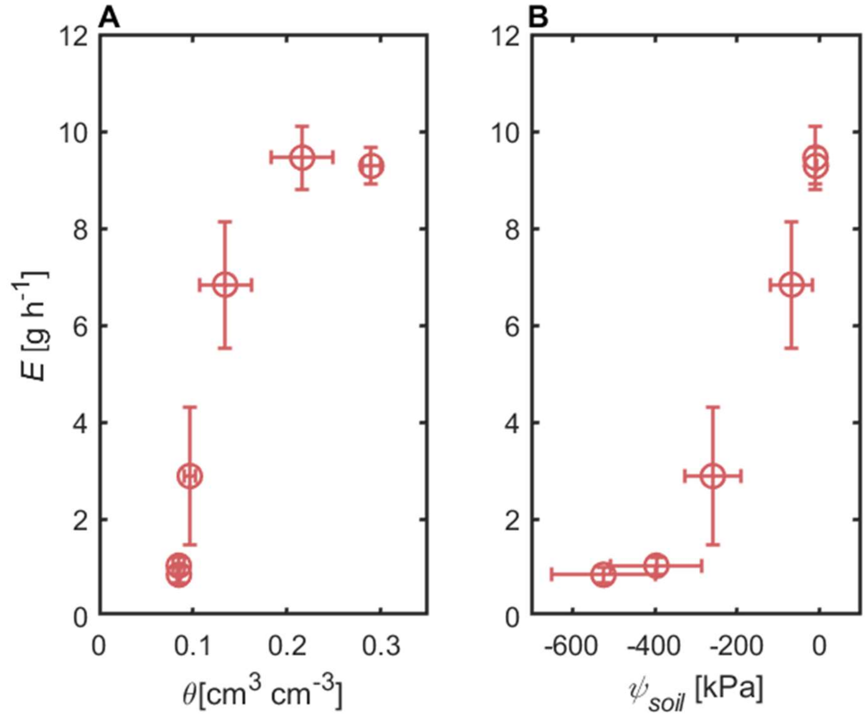

Fig. S4. Transpiration rate (*E*) of the entire canopy decreases during soil drying **A**) soil water content (θ), and **B**) soil water potential (ψ_{soil}).
